# Influence of Isothermal Aging on Microstructure and Shear Property of Novel Epoxy Composite SAC305 Solder Joints

**DOI:** 10.3390/polym15204168

**Published:** 2023-10-20

**Authors:** Peng Zhang, Songbai Xue, Lu Liu, Jianhao Wang, Hiroaki Tatsumi, Hiroshi Nishikawa

**Affiliations:** 1 College of Materials Science and Technology, Nanjing University of Aeronautics and Astronautics, Nanjing 210016, China; 2 Joining and Welding Research Institute, Osaka University, Ibaraki 5670047, Japan; 3 Institute of Intelligent Welding and Precision Manufacturing, Shanghai Jiao Tong University, Shanghai 200240, China

**Keywords:** epoxy composite SAC305 solder joint, thermal aging, reliability, microstructure, shear force

## Abstract

With the rapid iteration of microsystem integrated technology, the miniaturization of electronic devices requires packaging materials with higher reliability. In this work, the microstructure evolution and mechanical properties of novel epoxy composite SAC305 solder joints were studied after isothermal aging to evaluate the enhanced effect of epoxy addition. The thickness variation and morphological evolution of the interfacial layer were analyzed. The results showed that, as the aging time was prolonged, the Cu_6_Sn_5_ interfacial layer remarkably coarsened and Cu_3_Sn compounds formed between the Cu_6_Sn_5_ layer and Cu pad due to the continuous atomic diffusion. Compared with the monolithic joint, the epoxy composite SAC305 joints had a lower overall IMC growth rate during aging, closely related to the initial morphologies of the interfacial layers. The shear test results showed an apparent decrease in the shear forces of all the solder joints as the aging time increased. Nevertheless, because of the extra mechanical support provided by the epoxy layer, the epoxy composite joints demonstrated notably enhanced mechanical properties. After 1000 h aging treatment, the shear force of SAC305 joints containing 8 wt.% epoxy was 26.28 N, showing a 24.08% increase over the monolithic joint. Cu-Sn IMCs were detected on the shear fracture of the monolithic joint after 1000 h aging, indicating the fracture occurred near the interface and displayed a ductile/brittle mixed fracture. Concerning the epoxy composite joints, cracks were still initiated and extended within the solder bulk, demonstrating a noticeable enhancement in ductility due to the addition of epoxy.

## 1. Introduction

In recent decades, to meet the design requirements of continuous miniaturization and high performance for electronic devices, advanced electronic packaging technologies have been extensively developed and utilized in the electronic packaging industry [1,2,3]. Sn-based solder is the most commonly used packaging material today, which provides crucial mechanical support and electrical connection between substrates and chips or electronic components [4,5]. Owing to the toxicity of the Pb element, the application of Pb-containing solders is strictly restricted by WEEE and RoHS 2.0 directives [6]. To make up for this situation, some lead-free solder alloys, such as Sn-Ag-Cu, Sn-Cu and Sn-Bi alloys, were developed. Among these solder systems, Sn-Ag-Cu solder alloys have become the most promising alternative for traditional Pb-containing solder because of their ideal mechanical properties and superior creep resistance [7,8].

Meanwhile, with the arrival of the era of big data, 5G communication and AI technology have been rapidly developed [9]. The density of electronic components in integrated circuits (ICs) is rising, accompanied by a continuous reduction in size [10]. Considering high operation current density and decreased joint size, the reliability of micro-joints under thermal loads is inevitably affected [11]. Generally, the generation of a uniform and continuous interfacial IMC layer indicates a robust solder joint. However, these brittle IMCs, such as Cu_6_Sn_5_ and Cu_3_Sn, coarsen excessively under thermal loads, which may lead to the formation of structural defects and reliability degradation of the micro-joint [12,13]. During the aging process of the SnAgCu/Cu joint, a Cu_3_Sn IMC layer forms and overgrows in the solid-state reaction, with the simultaneous emergence of Kirkendall voids [14]. Wang et al. [15] attributed the reduced impact strength of the SAC305 solder bump to the thickened interfacial compounds and the coarsened grain of solder bulk during aging treatment.

With the increased thermal and mechanical loads that interconnected joints withstand, solders are expected to have higher thermal reliability [16,17]. Many researchers have reported that adding alloy elements (e.g., Ni, Ga, In, rare earth (RE)) and nanoparticles (e.g., Al_2_O_3_, SiC, CNTs, GNSs) to lead-free solder alloys is a feasible method [18,19,20]. It can effectively hinder interfacial reaction and the coarsening of interfacial IMCs, resulting in higher joint reliability. Among these reinforcements, carbon-based nanomaterials with low density and superior thermophysical properties have drawn significant attention. These were investigated by Jung et al. [21], who pointed out that Ag-MWCNTs could also improve the thermal and electrical performance of Sn-58Bi solder joints during thermal aging. However, considering issues such as high cost and the agglomeration of nanoparticles with high surface energy, solders containing additional alloy elements and second-phase particles face challenges in achieving widespread utilization [22].

In the past few years, owing to the excellent wetting and high bonding strength, the development and performance evaluation of Sn-based solder materials containing epoxy have been research hotspots [23,24,25]. Jung et al. [26] found that the addition of epoxy to Sn-58Bi solder lowered the activation energy for IMC growth and thus reduced the IMCs’ growth rate during aging, resulting in higher fracture energy and shear strength of the joint. Sung et al. [27] reported that the Sn-58Bi epoxy solder joint had better thermal shock reliability, which could withstand more cycles than the original solder joint. Our group [28] reported that, after undergoing 1000 thermal cycles or 1000 h humidity treatment, the epoxy layer on the joint surface continued to deliver a noticeable mechanical enhancement to the Sn-58Bi joint. In addition, high-performance epoxy composite Sn-3.0Ag-0.5Cu (SAC305) solder paste was developed in our previous study, and its spreadability, microstructure and shear performance were investigated [29]. To explore the possibility of its application in high-density packaging, a thermal aging test was conducted in this work. The microstructure and morphology evolution of the interfacial layer during isothermal aging were analyzed. A shear test was performed to address the influence of epoxy addition on the mechanical property, and then the fracture morphologies were observed.

## 2. Materials and Methods

The monolithic SAC305 solder paste was fabricated using solder powders (25–45 μm diameter) and no-clean flux, which were supplied by Hoerson Electronics Technology Company Limited, Shenzhen, China. In this study, methyltetrahydrophthalic anhydride (MTHPA; Guangzhou Zhonggao Chemical Company Limited, Guangzhou, China) served as the curing agent for E51 epoxy (Nantong Xingchen Synthetic Material Company Limited, Nantong, China), and the ratio of E51 epoxy to the curing agent was 100:85. In addition, trace amounts of 2,4,6-Tris-(dimethylaminomethyl)phenol (DMP-30; Guangzhou Zhonggao Chemical Company Limited, Guangzhou, China) were incorporated as an accelerator to expedite the curing process of the E51 epoxy. The monolithic SAC305 solder paste was mixed with the E51 epoxy curing system (including curing agent and accelerator) in proportion to prepare epoxy composite SAC305 solder pastes. The mixing process described above is performed using a solder paste mixer (MIX500D2, RobotDigg, Shanghai, China) with a rotation rate of 600 rpm, and the stirring time is 5 min. During the high-speed rotation process, the components of the composite solder paste were uniformized, and, simultaneously, the deaeration of the epoxy was accelerated. For the sake of brevity, the prepared SAC305 solder pastes containing 4 wt.%, 8 wt.% and 12 wt.% epoxy resin curing system are denoted by SAC305-4ER, SAC305-8ER and SAC305-12ER, respectively. The thermal behaviors of the monolithic SAC305 solder paste and epoxy composite SAC305 solder pastes were investigated by differential scanning calorimeter (DSC; STA 449 F3, Netzsch, Selb, Germany). The heating rate was set as 10 °C/min from 30 °C to 300 °C.

**Figure 1:**
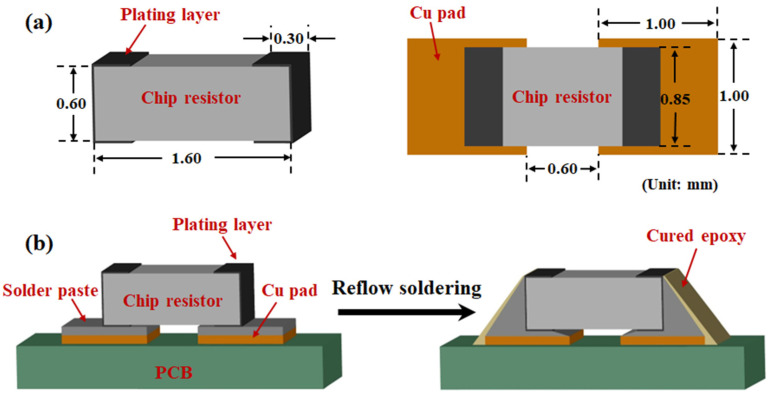
Schematic diagram of (**a**) dimensions of the chip resistor and Cu pad and (**b**) the preparation process of epoxy composite solder joint during the reflow soldering.

Next, 0603 chip resistors were soldered to OSP-Cu pads on a printed circuit board (PCB) using the above SAC305 solder pastes through reflow soldering. The parameters of the 0603 chip resistors and the Cu pad are shown in Figure 1a. The total time of the reflow process was 5 min, with a peak temperature of 250 °C. Figure 1b presents the schematic diagram of the preparation process of the epoxy composite solder joint during reflow soldering. More details about the specimen preparation were described in our previous study [29]. After reflow soldering, monolithic SAC305 and epoxy composite SAC305 solder joints were aged at 150 °C for 300 h, 600 h and 1000 h. The board-level isothermal aging test was conducted by placing the reflowed PCB into a constant temperature oven set at 150 °C with an ambient atmosphere. For each aging condition, 10 specimens of each solder joint composition were prepared for subsequent shear test and microstructure observation.

The macroscopic morphologies of the aged joints were observed to analyze the macro-appearance change in the cured epoxy. In order to investigate the cross-section microstructure, the specimens were cut by a gravity-fed precision sectioning machine (IsoMet Low Speed, Buehler, Lake Bluff, IL, USA). Subsequently, with the aid of a semi-automatic grinding and polishing machine (Tegrapol-15, Struers, Ballerup, Denmark), the specimens were initially polished by #400, #1200 and #2000 SiC sandpapers and 3 μm and 1 μm Al_2_O_3_ grinding paste, and acidic colloidal silica suspension was used for the final polishing. The top view of the interfacial IMCs was observed after removing the solder above the interface layer with thin nitric acid. The cross-sectional microstructure and top-view morphology of the IMC layer were analyzed by SEM (JSM-IT200, JEOL, Tokyo, Japan) and the attached EDS, and then the thickness and grain size of the interfacial IMC were measured by image-J software (version: 1.54d).

The shear forces of the aged solder joints were measured using a micro-joint strength tester (STR1000, Rhesca, Tokyo, Japan) according to Japanese Industrial Standard JIS Z3198-7 [30]. The shearing tip operated at a speed of 2 mm/min, with a shear height of 100 μm. At least five solder joint specimens were tested for each condition, and their average value was reported. After the shear test, the fracture surfaces were observed by SEM and EDS to analyze the fracture behaviors of the solder joints.

## 3. Results

### 3.1. Thermal Behaviors of the Solder Pastes

**Figure 2:**
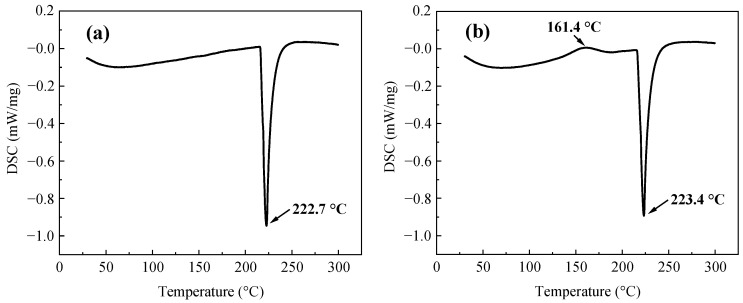
DSC result of (**a**) the monolithic SAC305 solder paste and (**b**) SAC305-8ER solder paste.

Figure 2 shows the DSC curves of the thermal behaviors of the monolithic SAC305 and SAC305-8ER solder pastes. It can be seen from Figure 2a that there was an obvious endothermic peak at 222.7 °C, corresponding to the melting characteristics of the SAC305 solder alloy. For the SAC305-8ER solder paste, the liquid peak of the solder alloy was 223.4 °C, which was nearly identical to the monolithic solder paste. In addition, an exothermic peak was detected before the melting of the solder alloy in Figure 2b, which indicated the curing behavior of the added epoxy resin. However, the heating rate during reflow soldering is significantly higher than that of the DSC test, and thus the peak temperature of the curing reaction will be increased, while the melting temperature of solder alloy is almost constant [31].

### 3.2. Microstructure Observation

**Figure 3:**
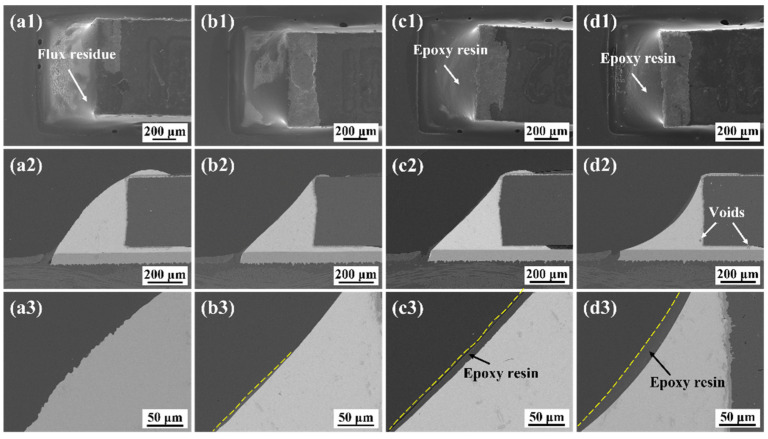
SEM images of the solder joints after reflow soldering: (**a1**–**a3**) monolithic SAC305; (**b1**–**b3**) SAC305-4ER; (**c1**–**c3**) SAC305-8ER; (**d1**–**d3**) SAC305-12ER.

Figure 3 reveals the macroscopic morphologies and microstructure of the as-reflowed monolithic SAC305 and epoxy composite SAC305 solder joints. It can be seen that an excellent metallurgical connection was established between the 0603 resistor and the Cu pad. During the reflow process, the added epoxy moved to the surface of the molten solder alloy due to its high fluidity and low density and then formed thermoplastic production by a curing reaction. As shown in Figure 3c1, with the addition of 8 wt.% epoxy, it can be observed that a continuous epoxy surrounding layer adhered to the joint surface. However, some voids were generated in the solder matrix if excessive epoxy was added, as shown in Figure 3d2. The void formation can be attributed to the organics residue that failed to evacuate from the solder matrix during the reflow process.

**Figure 4:**
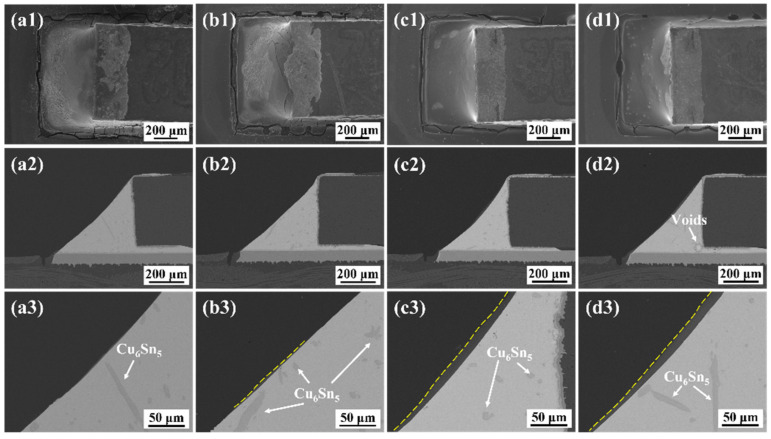
SEM images of the solder joints aged at 150 °C for 1000 h: (**a1**–**a3**) monolithic SAC305; (**b1**–**b3**) SAC305-4ER; (**c1**–**c3**) SAC305-8ER; (**d1**–**d3**) SAC305-12ER.

Figure 4 shows the macro-appearance and microstructure of the monolithic SAC305 and epoxy composite SAC305 solder joints aged for 1000 h. Given that the epoxy layer remained intact, it could still provide extra mechanical support for the aged joints. Moreover, some coarsened dark grey phases could be detected in the solder matrix at high magnification and were identified as Cu_6_Sn_5_ IMC by EDS analysis.

**Figure 5:**
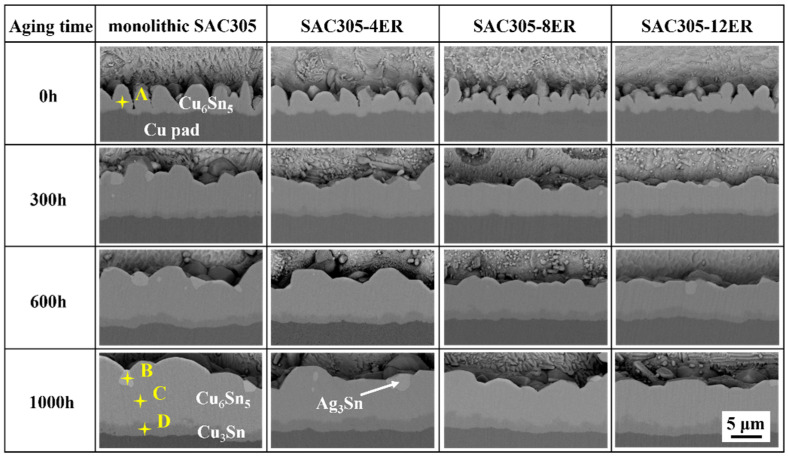
Cross-sectional microstructure of monolithic SAC305 and epoxy composite SAC305 solder joints aged for various aging times.

**Figure 6:**
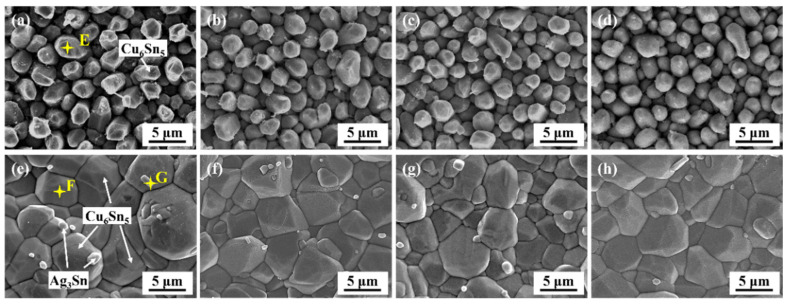
Top-view images of interfacial IMCs of the joints after aging for (**a**–**d**) 0h and (**e**–**h**) 1000 h: (**a**,**e**) monolithic SAC305; (**b**,**f**) SAC305-4ER; (**c**,**g**) SAC305-8ER; (**d**,**h**) SAC305-12ER.

As Figure 5 shows, a continuous scallop-like interfacial layer formed above the Cu pad after the reflow soldering. Based on the EDS analysis shown in Table 1 and the previous study by [32], the compound could be confirmed as Cu_6_Sn_5_ IMC. It should be noticed that the IMC layer thicknesses of all the as-soldered joints were similar because they had undergone the same reflow process. Nevertheless, the interfacial layer of the epoxy composite joint exhibited a relatively flat and smooth morphology relative to the monolithic joint. This phenomenon can also be revealed in the top-view microstructure of the interfacial IMCs of the as-soldered joints. As shown in Figure 6a, there were visible gaps between the interfacial Cu_6_Sn_5_ particles of the as-soldered monolithic joint, and the height difference between these particles was large. Nevertheless, the interfacial IMCs of the as-soldered epoxy composite joint gradually exhibited a homogeneous morphology with reduced grain size, as presented in Figure 6b–d. The interfacial IMC showed an average grain size of 3.7 μm, 3.0 μm, 2.7 μm and 2.6 μm for the monolithic SAC305, SAC305-4ER, SAC305-8ER and SAC305-12ER solder joints, respectively.

During the manufacturing process of soldering materials, organic acids and their anhydrides are commonly used as activators in the flux to enhance the interaction between the solder and the substrate [33,34]. The observed changes in morphology mentioned above could be attributed to the anhydride-type curing agent present in the epoxy resin curing system. The agent near the Cu substrate assisted in removing the oxides of the Cu substrate during the soldering heating process [29]. Consequently, while serving as a curing agent, it also reduced the interfacial tension between the molten solder alloy and the copper substrate, facilitating the wetting process. As a result, the Cu diffusion from the substrate and interfacial IMC dissolution were enhanced at the initial stage of the soldering process. Yu and Wang reported [35] that the dissolution would facilitate the formation of a smooth and uniform interfacial layer with little roughness. It was supposed that more nucleation sites were provided with the epoxy addition and the growth between adjacent IMC grains was equivalent, resulting in a smoother IMC morphology. In summary, for the epoxy composite SAC305 solder joint, the IMCs produced by interfacial reactions were more homogeneous with lower roughness. A similar phenomenon was observed by Zhang et al. [36]. It was found that, with the assistance of ultrasonic waves, the oxidation film was effectively removed and the Cu diffusion was promoted, leading to a fine and homogeneous interfacial layer.

During the subsequent aging, the interfacial IMC layer gradually thickened and became flatter for a given joint type, accompanied by the emergence of a new IMC layer between the Cu_6_Sn_5_ IMC and Cu substrates. With regard to the EDS elemental analysis (Point D in Figure 5 and Table 1) and the Cu-Sn phase diagram [37], the new Cu-Sn interfacial compound exhibited an atomic ratio of 3:1, which was supposed to be a Cu_3_Sn IMC. After the same aging time, it could be observed from the cross-section images that the joints bearing epoxy had thinner IMC layers than the monolithic joints. Simultaneously, as depicted in Figure 6e–h, following a 1000 h aging process, the Cu_6_Sn_5_ IMC grain obviously coarsened, accompanied by the detection of some minute white Ag_3_Sn IMC embedded on the Cu_6_Sn_5_ grain surface. The Cu_3_Sn compound was covered by the Cu_6_Sn_5_ IMC layer, and was hardly observed. Moreover, the IMC grain sizes of the SAC305 joints bearing 4%, 8% and 12% were 12.0 μm, 9.2 μm and 10.2 μm, respectively, which were smaller than those of the monolithic joints (18.9 μm). The following section will discuss and analyze the variation in thickness and the coarsened behaviors of the interfacial layer.

### 3.3. IMC Growth Kinetics

**Figure 7:**
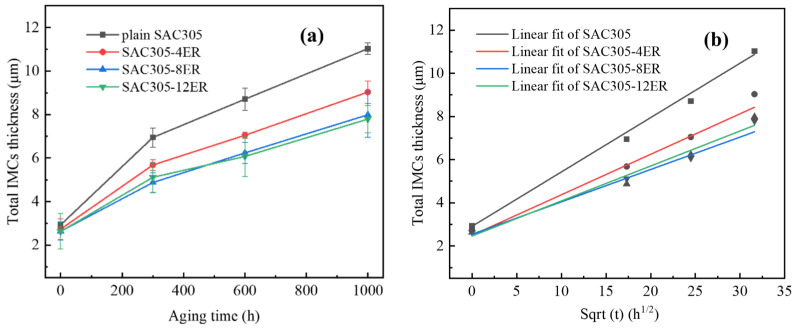
(**a**) Variation in the interfacial layer thickness of the monolithic SAC305 and epoxy composite SAC305 solder joints over thermal aging time; (**b**) fitted curve.

Figure 7a illustrates the total thickness variation of the interfacial layer of the monolithic SAC305 and epoxy composite SAC305 solder joints for various aging times. It is evident that, as the aging time increased, the total thickness of all the aged joints showed a significant increase. After undergoing a 1000 h aging process, the interfacial layer of the monolithic SAC305 solder joint exhibited the highest thickness of 11.03 μm, while those solder joints bearing 4, 8 and 12 wt.% epoxy resins had thinner IMC thicknesses, with 9.03 μm, 7.99 μm and 7.79 μm, respectively.

It is widely recognized that the atomic diffusion mechanism dominates IMC growth at the interface of the solder bulk/Cu pad during aging, which can be expressed as [38,39]:

$$x_{t}=x_{0\;}+\sqrt{Dt}$$

where *x_t_* is the thickness of the interfacial IMC layer after *t* h aging, *x*_0_ is the primary IMC thickness before aging and *D* is the diffusion coefficient. Based on the above formula, there exists a linear relationship between *x_t_* and the square root of *t*. Figure 7b shows the linear fitted curves between IMC thickness (*x_t_*) and the square root of the aging time (*t*), and the fitting data are listed in Table 2. By calculating the slope of each curve, it can be concluded that the epoxy composite joints have smaller growth coefficients than the monolithic joints, and that the joints bearing 8 wt.% and 12 wt.% epoxy resins have the smallest values. Kim et al. [26] reported a similar research result regarding the IMC growth behavior of an Sn-58Bi epoxy solder joint. They found that the epoxy-enhanced Sn-58Bi solder joint demonstrated a lower IMC growth rate than the monolithic Sn-58Bi solder joint and the former had a higher activation energy for IMC growth.

During the reflow process, the primary chemical reaction conducted between the melted solder alloy and Cu pad was the nucleation and growth of Cu_6_Sn_5_ IMC, as described below [40]:

$$6\mathrm{Cu}+5\mathrm{Sn}\;\to \;Cu_{6}Sn_{5}$$

**Figure 8:**
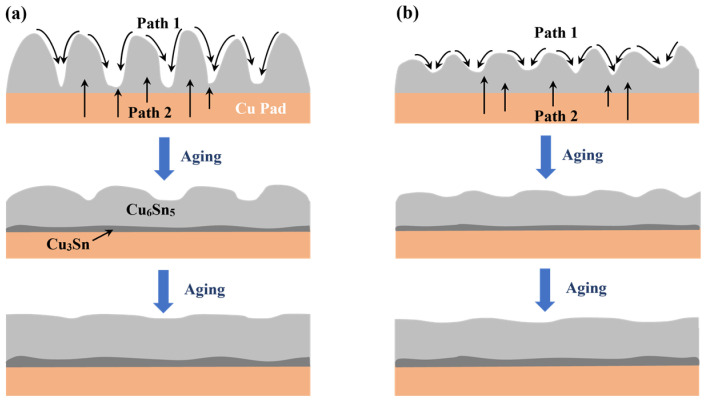
Schematic diagram of interfacial layer growth during aging treatment: (**a**) monolithic SAC305 solder joint; (**b**) epoxy composite SAC305 solder joint.

In the subsequent solid-state aging, the Cu_6_Sn_5_ gradually thickened because of the continuous diffusion of Cu and Sn atoms. It has previously been mentioned that, at room temperature or during solid-state thermal aging, Cu can move into Sn and Cu_6_Sn_5_ IMC by interstitial diffusion, which is easier than the movement of Sn to Cu, dominated by lattice diffusion [41]. As illustrated in Figure 8, the Cu flux engaged in the interfacial reaction primarily originated from two parts. Firstly, the Cu flux from the scallop peak to the valley (Path 1) was propelled by the curvature effect to reduce the surface energy of the scallop-like interfacial IMCs. The other is the Cu diffusion flux from the Cu substrate (Path 2) [42]. The transformation of morphology from scallop type to layer type can be attributed to the short diffusion path existing between the scallop valley and the Cu pad. Compared with the epoxy composite joints, the interfacial layer of the monolithic joint was more uneven and thus thickened rapidly at the valley in the subsequent aging. Therefore, although the thickness of the as-soldered joints was similar, the interfacial IMC growth of the monolithic joint was faster. This phenomenon was more pronounced for the joints with higher epoxy content. Deng et al. [43] investigated the influence of the initial morphology and thickness of the interfacial layers on the IMC growth of Sn-Ag/Cu joints during isothermal aging. It was also pointed out that, due to the shorter diffusion path in the scallop valleys, the joint with a scallop-like IMC layer had a higher IMC growth rate during aging compared to the joint with a flat interfacial IMC layer.

As the aging time increased, the formation of the Cu_3_Sn compound took place between the Cu_6_Sn_5_ IMC layer and the Cu pad, resulting from the gradual consumption of Cu_6_Sn_5_ IMC, as depicted below [44]:

$$Cu_{6}Sn_{5}+9\mathrm{Cu}\;\to \;5Cu_{3}\mathrm{Sn}$$

With the continuous atomic diffusion during aging, the IMC layers of all the joints thickened, and the top-view images indicated that the IMC grain sizes increased, accompanied by the planarization process.

### 3.4. Shear Force

**Figure 9:**
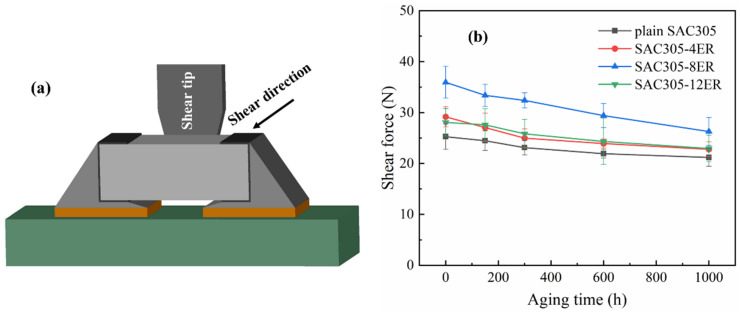
(**a**) Schematic diagram of shear test; (**b**) shear forces of monolithic SAC305 and epoxy composite SAC305 solder joints during thermal aging.

Long-term aging will lead to the reliability degradation of tin-based solder joints because of the accelerated atomic diffusion and stress concentration. The shear test is the most direct means of assessing joint reliability. Figure 9 displays the schematic diagram of the shear test and the shear forces of the monolithic SAC305 and epoxy composite SAC305 solder joints after isothermal aging. With the extension of aging time, all the solder joints displayed a gradual reduction in the shear forces. Nevertheless, the epoxy composite joints exhibited higher shear forces compared to the monolithic joint following an equivalent aging period. After 1000 h aging, the shear forces of SAC305 composite joints containing 4%, 8% and 12% epoxy resin were 22.78 N, 26.28 N and 22.94 N, which were still 7.55%, 24.08% and 8.31% higher than those of the monolithic joint, respectively.

The decrement in the mechanical property of tin-based solder joints can be mainly ascribed to the coarsening of the solder bulk and the thickening of the brittle interfacial layer [15]. Significantly, the thickness of the interfacial layer plays a crucial role in determining joint reliability. When the thickness is beyond a particular value, the mechanical performances of solder joints will be obviously reduced [45]. In this study, the Cu-Sn interfacial layer gradually coarsened and thickened during isothermal aging. Therefore, stress concentration was more likely to be generated near the brittle interfacial IMC layer, deteriorating the shear performance of the aged joints. In comparison to the SAC305-8ER solder joint, the SAC305-12ER solder joint had a similar IMC thickness, but its shear force was lower. This could be attributed to the excessive epoxy addition, which led to the generation of voids during reflow, consequently undermining the structural integrity of the joint.

### 3.5. Fracture Morphology

**Figure 10:**
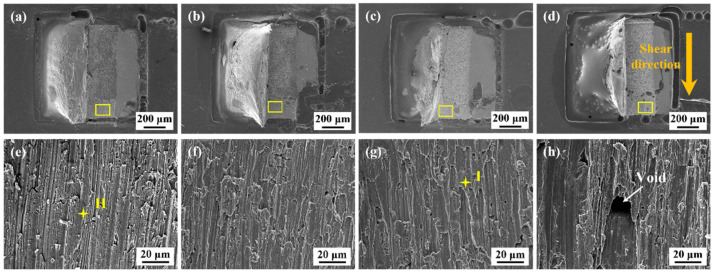
Fracture surfaces of the joints after reflow soldering: (**a**) monolithic SAC305; (**b**) SAC305-4ER; (**c**) SAC305-8ER; (**d**) SAC305-12ER; (**e**) enlarged image of the marked region in (**a**); (**f**) enlarged image of the marked region in (**b**); (**g**) enlarged image of the marked region in (**c**); (**h**) enlarged image of the marked region in (**d**).

To further determine the impact of epoxy addition on the shear performance of the solder joint, the fracture morphology was observed after the shear test. Figure 10 describes the fracture surfaces of the SAC305 joints after reflow soldering. As the epoxy content increased, epoxy resin gradually covered the joint surface and exhibited broken morphologies after the shear test. The corresponding fracture surfaces of all the as-soldered joints displayed characteristic shear fracture features. The fracture surface was uniform, and the EDS analysis in Table 3 (Point H and I) revealed that the composition mainly consisted of the Sn element. Thus, whether or not they contained epoxy resin, the fracture of all the as-soldered joints occurred inside the solder matrix and presented ductile. The fractured epoxy morphology revealed that it also undertook partial shear loads during the shear test and provided additional mechanical reinforcement. Nevertheless, when the epoxy content approached 12 wt.%, some voids in the solder matrix in the epoxy layer weakened the mechanical properties.

**Figure 11:**
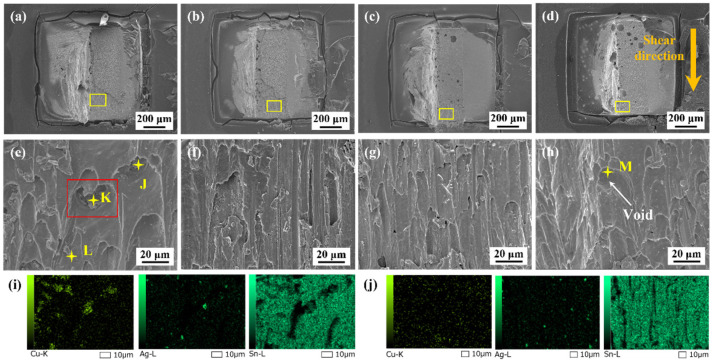
Fracture morphologies of the joints aged for 1000 h: (**a**) monolithic SAC305; (**b**) SAC305-4ER; (**c**) SAC305-8ER; (**d**) SAC305-12ER; (**e**) enlarged image of the marked region in (**a**); (**f**) enlarged image of the marked region in (**b**); (**g**) enlarged image of the marked region in (**c**); (**h**) enlarged image of the marked region in (**d**); (**i**) EDS element mapping of (**e**); (**j**) EDS element mapping of (**g**).

**Figure 12:**
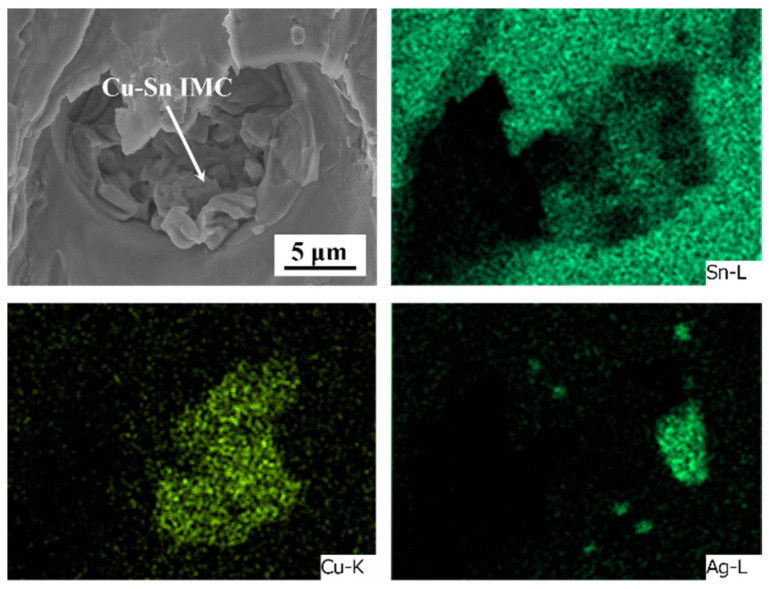
Enlarged image and its elemental mapping of the marked region in Figure 11e.

Figure 11 shows the fracture surfaces of the monolithic SAC305 and epoxy composite SAC305 solder joints aged for 1000 h. As presented in Figure 11e, the fracture morphology of the monolithic SAC305 solder joint displayed some shear dimples, and several pits were observed at the dimple bottom. The EDS element mapping showed that the Cu content in the pits was high, and the internal particles could be identified as a Cu-Sn IMC based on the EDS analysis results listed in Table 3. Figure 12 shows the magnified image of the marked region in Figure 11e, indicating that some Cu-Sn IMCs were exposed at the dimple bottom. This means that the crack propagated through the brittle interfacial layer. The thickness and morphology of the interfacial layers play a crucial role in the evolution of the fracture behaviors of the as-aged SAC305 solder joints. The interfacial IMCs exhibited rapid coarsening and flattening during aging, leading to pronounced stress concentration near the interface due to their inherent brittleness. Thus, the cracks were prone to nucleate and propagate near the interface under the external forces. It can be seen that, after isothermal aging, the fracture position gradually moved from the solder bulk towards the vicinity of the interface of the solder bulk/IMC layer, and that the fracture mode displayed a ductile/brittle mixed pattern. Wu et al. [46] also reported that, upon reaching a specific duration of aging, the fracture behaviors of a low-silver SnAgCu/Cu joint transformed from occurring within the solder bulk to taking place at the Cu-Sn IMC layer, due to the thermal expansion mismatch.

**Figure 13:**
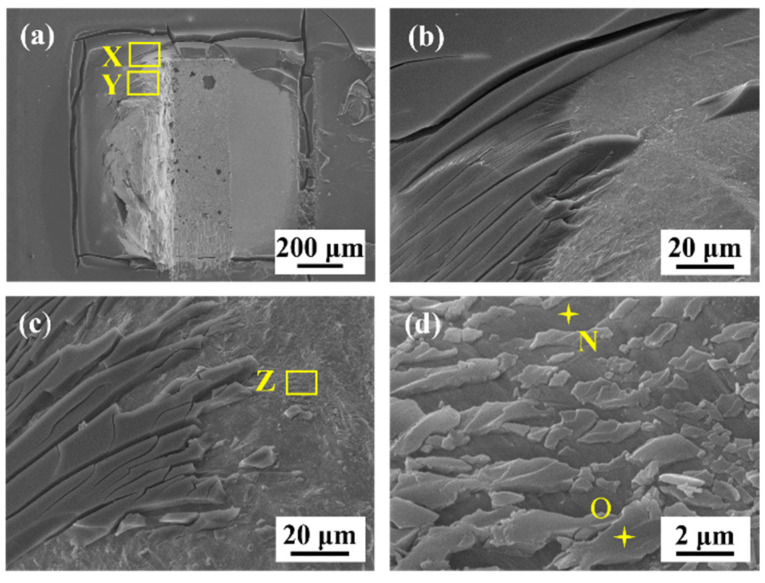
Fracture morphologies of SAC305-8ER solder joints aged for 1000 h: (**a**) macroscopic appearance; (**b**) magnified image of Area X; (**c**) magnified image of Area Y; (**d**) enlarged image of Area Z.

However, as presented in Figure 11b–d, the fracture still presented ductile characteristics for the epoxy composite joints, and the fracture location remained in the solder matrix. The ductility improvement could be ascribed to a lower IMC growth rate, which reduced the stress concentration caused by the excessive growth of brittle IMCs. More details are given in Figure 13 to observe the broken epoxy resin of the SAC305-8ER solder joint after fracture. It can be observed from the magnified image in Figure 13d that some broken epoxy residue (the light gray area, such as Point O) was attached to the joint surface (the dark gray area, such as Point N) after the shear test, which indicated the excellent adhesion between the epoxy layer and the solder surface.

## 4. Conclusions

In this study, an isothermal aging test was carried out to assess the thermal reliability of epoxy composite SAC305 solder joints. The impact of the epoxy addition on the interfacial growth behaviors, shear properties and fracture morphologies of the joints was analyzed. The following conclusions can be drawn:

1. After high-temperature storage for 1000 h, the macroscopic images revealed the epoxy layer on the joint surface remained intact without any noticeable defects. As the aging time was prolonged, the thicknesses and grain sizes of the interfacial IMC layer of all the joints increased, and the Cu_3_Sn layer emerged between the Cu_6_Sn_5_ layer and the Cu pad. However, the growth rate of the interfacial IMC layer of the epoxy composite joint was lower than that of the monolithic joint.
2. The addition of epoxy resulted in a more uniform morphology of interfacial IMCs in the as-soldered joints, leading to higher thermal stability in the epoxy composite solder joints. With the addition of 4 wt.%, 8 wt.% and 12 wt.% epoxy, the growth coefficients of the IMC layer gradually decreased from 1.74 × 10^−17^ m^2^/s to 1.00 × 10^−17^ m^2^/s, 0.63 × 10^−17^ m^2^/s and 0.71 × 10^−17^ m^2^/s, respectively.
3. The shear forces of the epoxy composite joints were remarkably increased due to their thinner IMC layers and the extra bonding area created by the epoxy layer. After aging for 1000 h, the fracture location of the monolithic joint transformed from the solder bulk to the interface of the solder/IMC layer, which presented a ductile/brittle mixed fracture. However, the fracture mode of the epoxy composite joint remained almost unchanged after long-term aging, showing its better ductility.

## Figures and Tables

**Table 1:** EDS elemental analysis in Figure 5 and Figure 6 (at.%).

Points	Cu	Sn	Ag
A	55.67	44.33	-
B	-	25.93	74.07
C	52.95	47.05	-
D	75.69	24.31	-
E	56.10	43.90	-
F	54.65	45.35	-
G	-	29.69	70.31

**Table 2:** Curve fitting results of Figure 7b.

Solder	Linear Fitted Curve	D Value(m^2^/s)
Monolithic SAC305	$y=2.91+0.25\sqrt{t}$	1.74 × 10^−17^
SAC305-4ER	$y=2.50+0.19\sqrt{t}$	1.00 × 10^−17^
SAC305-8ER	$y=2.54+0.15\sqrt{t}$	0.63 × 10^−17^
SAC305-12ER	$y=2.46+0.16\sqrt{t}$	0.71 × 10^−17^

**Table 3:** EDS elemental analysis in the fracture surface (at.%).

Points	Cu	Sn	Ag	C	O
H	-	96.99	3.01	-	-
I	0.92	97.61	1.47	-	-
J	53.69	45.19	1.12	-	-
K	50.27	46.77	2.96	-	-
L	0.66	98.11	1.23	-	-
M	1.09	97.87	1.03	-	-
N	5.77	92.73	1.50	-	-
O	-	-	-	69.71	30.29

## Data Availability

The data presented in this study are available on request from the corresponding author.
